# Probiotic *Propionibacterium freudenreichii* MJ2 Enhances Osteoblast Differentiation and Mineralization by Increasing the OPG/RANKL Ratio

**DOI:** 10.3390/microorganisms9040673

**Published:** 2021-03-24

**Authors:** Jiah Yeom, Seongho Ma, Young-Hee Lim

**Affiliations:** 1 Department of Integrated Biomedical and Life Sciences, Graduate School, Korea University, Seoul 02841, Korea; intro56@naver.com (J.Y.); aktjdgh8@naver.com (S.M.); 2 School of Biosystems and Biomedical Sciences, Korea University, Seoul 02841, Korea; 3 Department of Laboratory Medicine, Korea University Guro Hospital, Seoul 08308, Korea

**Keywords:** *Propionibacterium freudenreichii*, osteoblast mineralization, osteoprotegerin, receptor activator of nuclear factor-κB ligand, surface proteins

## Abstract

Osteoblast differentiation is important for the development of bone and the maintenance of bone density. *Propionibacterium freudenreichii* is a probiotic with an anti-inflammatory property. The aim of this study was to investigate the enhancement effect of *P. freudenreichii* MJ2 (MJ2) isolated from raw milk on osteoblast differentiation, mineralization, and its signaling pathway. For in vitro and in vivo experiments, human fetal osteoblastic cell line hFOB 1.19 and an ovariectomized rat model were used, respectively. Expression levels of genes and proteins related to osteoblast differentiation and mineralization were measured by real-time polymerase chain reaction (qPCR) and Western blotting, respectively. Alizarin red S staining was performed to measure osteoblast mineralization. Heat-killed MJ2 (hkMJ2)-treated cells showed significantly increased osteoblast differentiation via an increase in the osteoprotegerin (OPG)/receptor activator of nuclear factor-κB ligand (RANKL) ratio and significantly increased osteoblast mineralization by stimulating the expression of bone morphogenetic protein 2 and runt-related transcription factor 2. Additionally, oral administration of live or heat-killed MJ2 to ovariectomized rats inhibited osteoporosis-induced bone loss. Specifically, surface proteins isolated from MJ2 promoted osteoblast differentiation and mineralization. In conclusion, MJ2 enhanced osteoblast differentiation and mineralization through the OPG/RANKL signaling pathway and the effective component of MJ2 might be its surface proteins.

## 1. Introduction

Bone is a rigid tissue found only in vertebrates. Bones mainly play roles in structural support for mechanical functions, protection of internal organs, provision of an environment for hematopoiesis, and accumulation of minerals such as calcium and phosphorus [1]. Bone consists of osteoblasts, osteoclasts, and osteocytes. Osteoblasts, a bone-forming cell type, are derived from bone marrow mesenchymal stem cells and produce the bone extracellular matrix. Conversely, osteoclasts, a bone-resorbing cell type, are derived from bone marrow hematopoietic stem cells and have an erosive action by secreting proteolytic enzymes. Osteocytes are a long-lived cell type found in mature bone tissue, which are derived from mature osteoblasts and no longer function in bone formation [1].

In general, osteoblasts gradually change their shape as they differentiate, and finally have a cuboidal form and constitute the lining of bone surfaces [2]. They are responsible for the production of various components of the bone matrix. Therefore, osteoblasts are an obvious target to strengthen the bone structure. Osteoblast differentiation is regulated by various secreted factors, including bone morphogenetic proteins (BMPs), transforming growth factor-β (TGF-β), and fibroblast growth factors (FGFs), through various signaling pathways involving the BMP-2/runt-related transcription factor 2 (RUNX2) pathway, hedgehog signaling, Notch signaling, WNT signaling, and FGF signaling. Of these pathways, the BMP-2/RUNX2 pathway is modulated by BMPs and stimulates osteoblast-specific transcriptional factors including RUNX2. BMPs, which are members of the TGF-β superfamily, play major roles in embryonic development and skeletal formation [3]. RUNX2 is considered to be the predominant factor that mediates osteoblastic differentiation. This transcription factor has the ability to arbitrate the convergence of osteogenic pathways [4] and contributes to upregulating the expression of genes related to osteoblast differentiation, such as alkaline phosphatase (*ALP*), osterix (*OSX*), and fibronectin (*FN*). RUNX2 increases the osteoprotegerin (*OPG*)/receptor activator of nuclear factor-κB ligand (*RANKL*) ratio that is an important indicator of osteoblast differentiation. ALP is a common cellular protein and a crucial enzyme for cellular activity and early osteoblastic differentiation [4]. OPG inhibits osteoclast development and differentiation by interfering with the combination of RANKL and receptor activator of nuclear factor-κB (RANK) [5].

Throughout life, bone repeats the cycle of formation and breakdown, driven by osteoblasts and osteoclasts, respectively. The repeated cycle is exquisitely regulated, which is important for bone health. Thus, when this balanced system collapses, various bone diseases can occur [6,7]. Osteoporosis is a common bone disease in which bone becomes weaker and is likely to break because of a decrease in bone mass and structural deterioration. Reduction of bone mass is due to destruction of the balance between osteoblasts and osteoclasts, which results in more degradation of bone by osteoclasts than formation of bone by osteoblasts [1,8]. The risk of developing osteoporosis naturally increases with age. In addition to age, gender, smoking, and lifestyle are other risk factors. Particularly in postmenopausal women, bone mass declines rapidly because of the decrease in estrogen from menopause [9]. The prevalence of osteoporosis is increasing as the numbers of elderly people increase worldwide, which has become a serious global health problem. For osteoporosis treatment, many drugs have been developed, but they have side effects such as stomach upset, heartburn, and nausea [10,11]. The most common therapy for osteoporosis is the female hormones estrogen and progesterone. However, their well-known side effect is an increase in breast cancer risk [12]. Therefore, drugs without side effects or with fewer side effects should be developed. In addition to calcium and vitamin D, functional food ingredients such as phytoestrogens have been developed to reduce the risk of osteoporosis [13,14]. Recently, probiotics that prevent and improve osteoporosis have received increasing attention [15,16]. *Propionibacterium freudenreichii* is a probiotic bacterium that is generally recognized as safe. *P. freudenreichii* is used as a cheese starter and produces nutraceuticals, such as vitamins B12, B2, and K, and has bifidogenic activity that is characterized by enhancing the growth of bifidobacteria [17]. *P. freudenreichii* improves a dextran sulfate sodium-induced acute colitis rat model by stimulating MUC2 expression [18]. It also increases the mRNA expression levels of *dbl-1*, *tig-2*, *sma-3*, and *rnt-1* in *Caenorhabditis elegans* [19]. Among them, *dbl-1* and *tig-2* genes are homologs of vertebrate *BMP-5* and *BMP-8* genes, respectively, which function in the development of cartilage and bone [20]. Additionally, the *sma-3* gene is a homolog of the vertebrate *BMP-5* gene and the *rnt-1* gene is a homolog of human RUNX family genes involved in regulation of osteoblast proliferation and differentiation. On the basis of these previous reports, we hypothesized that the *P. freudenreichii* MJ2 strain might contribute to osteoblast differentiation and bone formation. In this study, the effect of *P. freudenreichii* MJ2 on bone health was investigated by focusing on its effect on osteoblast differentiation and mineralization as well as its signaling pathway using the human fetal osteoblast cell line hFOB 1.19, which is a typical human osteoblast cell model for in vitro studies such as human osteoblast differentiation and physiology [21], and ovariectomized (OVX) rats.

## 2. Materials and Methods

### 2.1. Materials

Human fetal osteoblast cell line hFOB 1.19 (ATCC CRL-11372) was purchased from the American Type Culture Collection (Manassas, VA, USA). Dulbecco’s modified Eagle’s medium/Nutrient Mixture Ham’s F-12 (DMEM/F12), fetal bovine serum (FBS), and penicillin/streptomycin were obtained from HyClone (Logan, UT, USA). 3-[4,5-Dimethylthiazol-2-yl]-2,5-diphenyltetrazolium bromide (MTT) was purchased from Amresco (Solon, OH, USA). p-Nitrophenyl phosphate (pNPP) and alizarin red S (3,4-dihydroxy-9,10-dioxo-2-antharacenesulfonic acid sodium salt) were purchased from Sigma (St. Louis, MO, USA). Cetylpyridinium chloride monohydrate was obtained from Samchun Chemical (Seoul, Korea). Recombinant human Noggin protein was purchased from R&D Systems (Minneapolis, MN, USA). Dimethyl sulfoxide (DMSO) was purchased from DAEJUNG (Siheung, Korea).

### 2.2. Cell Culture

hFOB 1.19 cells were grown in DMEM/F12 with 10% FBS, 100 U/mL penicillin, and 100 μg/mL streptomycin at 37 °C with 5% CO_2_. The medium was changed every 3 days. hFOB 1.19 cells under 10 passages were used in this study. The experiments were processed with cells with 80–90% confluence. Considering the ATCC recommendation and previous studies [22,23], the medium was not supplemented with ascorbic acid, beta-glycerophosphate, and dexamethasone in this study. The cells were seeded at a density of 2.5 × 10^4^ cells/mL and starved in serum-free DMEM/F12 medium for 24 h to synchronize the cell cycle.

### 2.3. Isolation of Propionibacterium freudenreichii MJ2 and Preparation of Heat-Killed P. freudenreichii MJ2

The *P. freudenreichii* MJ2 strain used in this study was isolated from non-pasteurized raw milk obtained from a local farm. Raw milk was serially diluted and plated on yeast extract lactate agar (YELA) that consisted of 1% tryptone, 1% yeast extract, 0.025% K_2_ HPO_4_, 0.005% MnSO_4_, 1% sodium lactate, and 1.5% agar (adjusted to pH 7.0 ± 0.05) [24]. After incubation at 30 °C under anaerobic conditions for 7 days, the strain was isolated after subculturing single colonies. Then, using 16S rRNA gene sequencing, the *P. freudenreichii* strain was identified and designated as *P. freudenreichii* MJ2. The strain (KCCM12272P) was deposited in the Korean Culture Center of Microorganisms. *P. freudenreichii* MJ2 was grown in reinforced clostridial medium (RCM) and cultured at 30 °C in an anaerobic conditioned chamber (GasPak™ EZ container system; BD, Franklin lakes, NJ, USA). After 48 h of incubation, bacterial cells were harvested by centrifugation at 3000× *g* for 10 min and then washed twice with phosphate-buffered saline (PBS). Pelleted bacteria were diluted to 1 × 10^9^ CFU/mL and killed by heating at 100 °C for 30 min. *P. freudenreichii* MJ2 was confirmed to not grow after heat treatment. *Lactobacillus plantarum* (newly classified as *Lactiplantibacillus plantarum*) [25] KACC15357 was purchased from the Korean Agricultural Culture Collection (Wanju, Jeollabuk-do, Korea) and was cultured in De Man, Rogosa, and Sharpe (MRS, Oxoid Ltd., Hampshire, United Kingdom) broth under anaerobic conditions at 37 °C for 24 h.

### 2.4. Measurement of Cell Viability

hFOB 1.19 cells were seeded in 96-well plates at a density of 2.5 × 10^4^ cells/mL. After overnight incubation at 37 °C, the cells were starved in a serum-free DMEM/F12 medium for 24 h and treated with heat-killed *P. freudenreichii* MJ2 (hkMJ2) (1 × 10^6^, 1 × 10^7^, or 1 × 10^8^ cells/mL) for 14 days. The medium was replaced every 3 days. The medium was aspirated and 0.5 mg/mL MTT were added. After incubation at 37 °C for 1 h, the supernatant was aspirated and the formazans were dissolved in DMSO for 1 h. To remove the precipitated high dose of hkMJ2, the plates were centrifuged by 3000× *g* for 5 min and then only the supernatant was collected. The absorbance was measured at 540 nm using a SpectraMax 340PC384 plate reader (Molecular Devices, Sunnyvale, CA, USA). Cell viability (%) was calculated as the percentage relative to the negative control group.

### 2.5. Alkaline Phosphatase (ALP) Activity Assay

hFOB 1.19 cells were seeded in 12-well plates at a density of 2.5 × 10^4^ cells/mL. After incubation at 37 °C overnight, the cells were starved in serum-free medium for 24 h and then treated with hkMJ2 (1 × 10^6^, 1 × 10^7^, or 1 × 10^8^ cells/mL) for 14 days. For the inhibition assay, the cells were cotreated with 100 ng/mL Noggin that is involved in the development of bones. The medium was replaced every 3 days. Then, the cells were washed with PBS and lysed in buffer that consisted of 10 mM Tris-HCl (pH 7.5), 1 mM MgCl_2_, and 0.2% Triton X-100. Cell suspensions were placed in ice under agitation and then centrifuged at 2500× *g* for 10 min at 4 °C. The supernatants were collected and each sample (50 µL/well) was loaded into a 96-well plate. pNPP substrate solution (50 μL) was added in the each well. After gentle mixing, the plate was incubated at room temperature for 30 min. The enzymatic reaction was stopped by adding 50 μL NaOH and absorbance was measured at 405 nm using the SpectraMax 340PC384 plate reader. The relative ALP activity was normalized to the total protein concentration quantified by a Bradford assay (Biorad, Hercules, CA, USA).

### 2.6. Quantitative Real-Time Polymerase Chain Reaction (qPCR)

hFOB 1.19 cells were seeded in 6-well plates at a density of 2.5 × 10^4^ cells/mL. After overnight incubation at 37 °C, the cells were starved in serum-free medium for 24 h and then treated with hkMJ2 (1 × 10^6^, 1 × 10^7^, or 1 × 10^8^ cells/mL) for 14 days. For the inhibition assay, the cells were cotreated with 100 ng/mL Noggin. The medium was replaced every 3 days. Total RNA was extracted using Ribo-Ex reagent (GeneAll Biotechnology, Seoul, Korea) in accordance with the manufacturer’s instructions. Extracted total RNA was quantified by a NanoDrop ND-1000 spectrophotometer (Thermo Scientific, Wilmington, DE, USA) and then converted to cDNA with a RevertAid First Strand cDNA Synthesis kit (Thermo Scientific). qPCR was performed using a Kapa SYBR Fast qPCR kit (Kapa Biosystems, Woburn, MA, USA) with the 7500 Fast Real-Time PCR System (Applied Biosystems, Foster City, CA, USA). The reaction was preheated to 95 °C for 10 min followed by 40 cycles at 95 °C for 15 s, 60 °C for 15 s, and 72 °C for 30 s. Glyceraldehyde-3-phosphate dehydrogenase (*GAPDH*) was used as the reference gene and the primer sequences used in this study are shown in Table 1. The primers were purchased from Bioneer (Seoul, Korea). Relative gene expression was quantified on the basis of equal amounts of RNA and the ΔCt (ΔCt = Ct_target gene_ − Ct_reference gene_) value was calculated. The ΔΔCt value was calculated by the following equation: ΔΔCt = (ΔCt_treated_ − ΔCt_untreated_). The normalized expression change was expressed as 2^−ΔΔCt^ (*GAPDH* control was set to 1) [26]. For animal samples, rat hind femur bone was excised, split into small pieces using liquid nitrogen, and homogenized in Ribo-Ex reagent for mRNA extraction. The supernatant was collected after centrifugation at 12,000× *g* for 10 min and the subsequent mRNA extraction and qPCR were performed as described above. For rat femur bone, *β-actin* was used as the reference gene and the primer sequences of *OPG*, *RANKL*, and *β-actin* are shown in Table 1.

### 2.7. Western Blotting

hFOB 1.19 cells were seeded in 60 mm dishes at a density of 2.5 × 10^4^ cells/mL. After overnight incubation at 37 °C, the cells were starved in a serum-free medium for 24 h and then treated with hkMJ2 (1 × 10^6^, 1 × 10^7^, or 1 × 10^8^ cells/mL) for 14 days. The medium was replaced every 3 days. Then, the cells were harvested and lysed for protein extraction using radioimmunoprecipitation assay (RIPA) buffer (Rockland Immunochemicals, Limerick, PA, USA) containing Halt™ protease inhibitor cocktail (Thermo Scientific). The total protein concentration was determined by the Bradford assay and proteins were denatured at 100 °C for 10 min. An equal amount of protein (20 μg) from each sample was separated by 10% sodium dodecyl sulfate-polyacrylamide gel electrophoresis. The separated proteins were transferred to a PVDF membrane (Millipore, Bedford, MA, USA) and blocked with 5% dry non-fat skim milk in Tris-buffered saline (TBS) with 0.05% Tween 20 (TBST) at room temperature for 2 h. The membrane was washed with TBST three times and then incubated with a primary antibody at 4 °C overnight. Antibodies against the endogenous control β-actin (1:5000 dilution, MA5-15739; Thermo Scientific), OPG (1:1000 dilution, GTX55734; Genetex, Irvine, CA, USA), RANKL (1:1000 dilution, GTX32834; Genetex), BMP2 (1:500 dilution, ab14933; Abcam), Smad1/5/9 (1:1000 dilution, ab66737; Abcam), and RUNX2 (1:1000 dilution, ab23981; Abcam) were used as primary antibodies. The membranes were washed with TBST three times and then incubated with a secondary antibody at room temperature for 1 h. A goat anti-mouse IgG (H+L) horseradish peroxidase-conjugated antibody (1:10,000 dilution, NCI1430KR; Thermo Scientific) was used for anti-β-actin detection and a goat anti-rabbit IgG (H + L) horseradish peroxidase-conjugated antibody (1:5000 dilution, NCI1460KR; Thermo Scientific) was used to detect the other proteins. After incubation, the membrane was washed with TBST three times and developed with a SuperSignal West Femto Maximum Sensitivity Substrate kit (Thermo Scientific). Images were obtained and quantified using the ImageJ Gel Analysis program (Softomic, Barcelona, Spain). For animal samples, rat hind femur was excised, and connective tissue and muscle were removed. Then, the femur bone was split into small pieces using liquid nitrogen and homogenized in RIPA buffer for protein extraction. After centrifugation at 13,000 rpm for 5 min, the supernatant was obtained, followed by protein denaturation. Western blotting was performed with primary antibodies against the endogenous control β-actin, OPG, and RANKL (1:1000 dilution, GTX108515; Genetex) as described above.

### 2.8. Two-Dimensional Electrophoresis (2-DE) and LC-MS/MS

2-DE was carried out as described previously [27]. Aliquots in sample buffer (7 M urea, 2 M thiourea, 4.5% 3-[(3-cholamidopropyl)dimethylammonio]-1-propanesulfonate, 100 mM dithioerythritol, and 40 mM Tris, pH 8.8) were applied to immobilized pH 3–10 nonlinear gradient strips (Amersham Biosciences, Uppsala, Sweden). Isoelectric focusing was performed at 80,000 Vh. The second dimension was analyzed on 9–16% linear gradient polyacrylamide gels (18 cm × 20 cm × 1.5 mm) at a constant 40 mA per gel for approximately 5 h. After protein fixation in 40% methanol and 5% phosphoric acid for 1 h, the gels were stained with Coomassie Brilliant Blue G-250 (Thermo Fisher) for 12 h. The gels were destained with H_2_O, scanned in a GS710 densitometer (Bio-Rad, Richmond, CA, USA), and converted into electronic files that then analyzed by the Image Master Platinum 5.0 image analysis program (Amersham Biosciences).

Nano-LC-MS/MS analysis was performed with an Easy n-LC (Thermo Fisher) and an LTQ Orbitrap XL mass spectrometer (Thermo Fisher) equipped with a nanoelectrospray source. The samples were separated on a C18 nanobore column (150 × 0.1 mm, 3 μm pore size; Agilent). Mobile phase A for LC separation was 0.1% formic acid and 3% acetonitrile in deionized water and mobile phase B was 0.1% formic acid in acetonitrile. The chromatography gradient was a linear increase from 0% B to 60% B in 9 min, 60% B to 90% B in 1 min, and 3% B in 5 min. The flow rate was maintained at 1.8 μL/min. Mass spectra were acquired using data-dependent acquisition with a full mass scan (380–1700 *m*/*z*), followed by 10 MS/MS scans. For MS1 full scans, the orbitrap resolution was 15,000 and the automatic gain control (AGC) was 2 × 10^5^. For MS/MS in the LTQ, the AGC was 1 × 10^4^. The mascot algorithm (Matrix Science, Boston, MA, USA) was used to identify peptide sequences in a protein sequence database. Database search criteria were as follows: taxonomy; Homo sapiens, fixed modification; carbamidomethylated at cysteine residues, variable modification; oxidized at methionine residues, maximum allowed missed cleavage; 2, MS tolerance; 10 ppm, MS/MS tolerance; 0.8 Da. The peptides were filtered with a significance threshold of *p* < 0.05.

### 2.9. Immunocytochemistry (ICC)

Cells were seeded on coverslips coated with 0.1% gelatin in a 24-well plate at a density of 1.25 × 10^4^ cells/mL. After overnight incubation at 37 °C, the cells were starved in serum-free medium for 24 h and then treated with hkMJ2 (1 × 10^6^, 1 × 10^7^, or 1 × 10^8^ cells/mL) for 14 days. The medium was replaced every 3 days. The cells were washed with PBS and then fixed with 4% paraformaldehyde at room temperature for 30 min. The cells were washed with PBS and then permeabilized with 0.1% Triton X-100 in PBS. Then, the cells were blocked with 10% normal donkey serum (NDS) at room temperature for 30 min. A primary antibody against RUNX2 (1:1000 dilution, ab23981; Abcam) in 2% NDS was diluted at 1:500 and applied at 4 °C overnight. The cells were washed with PBS and then incubated with a secondary antibody, goat anti-rabbit IgG DyLight 488 (1:1000 dilution, 35553; Thermo Scientific), in 2% NDS. Nuclei were counterstained with 4′,6-diamidino-2-phenylindole (Sigma) for 5 min and the coverslips were mounted with VECTASHIELD^®^ (Vector Laboratories, Burlingame, CA, USA). Images were obtained under a C1 plus confocal laser scanning microscope (Nikon, Tokyo, Japan).

### 2.10. Alizarin Red S Staining

Cells were seeded in a 12-well plate at a density of 2.5 × 10^4^ cells/mL. After overnight incubation at 37 °C, the cells were starved in serum-free medium for 24 h and then treated with hkMJ2 (1 × 10^6^, 1 × 10^7^, or 1 × 10^8^ cells/mL) for 21 days. The medium was replaced every 3 days. Then, the cells were washed with PBS and fixed with 4% paraformaldehyde at room temperature. The fixing solution was aspirated and the cells carefully washed with distilled water. Alizarin red S solution (2%) dissolved in distilled water (adjusted to pH 4.2) and filtered through a 0.22 µm syringe filter was prepared and the cells were stained with the solution at room temperature for 30 min in the dark. Then, the solution was aspirated and the cells were carefully rinsed with distilled water. Images were captured under an optical microscope and then the stained deposits were dissolved in 10% cetylpyridinium chloride. To quantify the levels of mineralization, the supernatants were transferred to a 96-well plate and the absorbance was measured at 562 nm.

### 2.11. Animal Model

Seventy-two female Sprague Dawley rats (9 weeks of age) weighing 170–190 g were purchased from Koatech (Pyeongtaek, Korea). They were maintained at 24 ± 1 °C with a 12 h light/dark cycle at 55% humidity. Animals were maintained with ad libitum access to a laboratory chow diet (Harlan diet 2018S, Koatech) and water. The experimental protocol was approved by the Korea University Institutional Animal Care and Use Committee (Approval No. KUIACUC-2020-0005). All experimental procedures were performed in accordance with the Guide for the Care and Use of Laboratory Animals (NIH Publication No. 85–23, 1996). After acclimation for 1 week, the rats were randomly divided into nine groups (n = 8 per group) (Table 2). Rats in the sham group were only subjected to an incision without removal of the ovaries and bilateral ovaries were removed from the other groups. The rats were treated with a daily oral injection of each test strains for 4 months and their weights were measured every week. For animal experiments, *Lactobacillus plantarum* KACC15357 was used as a positive reference strain. *L. plantarum* is known to prevent bone loss [28]. *L. plantarum* was cultured in MRS broth under anaerobic conditions at 37 °C for 24 h and the strain was prepared by the same procedure described above. Dead *P. freudenreichii* MJ2 was prepared by the same heat treatment procedure (100 °C for 30 min) described above.

### 2.12. Measurement of Bone Mineral Density (BMD)

High-quality 3D images of rat femurs were obtained using a micro-computed tomography (CT) system (SkyScan 1173, Konitch, Belgium) at a resolution of 20 μm, filter of 1.0 mm, exposure of 500 ms, voltage of 90 kV, and current of 88 μA. The images were reconstructed using Nrecon software (Ver. 1.7.4.2). Rat hind legs were scanned by dual-energy X-ray absorptiometry using an InAlyzer (Medikors, Seongnam, Korea) at a resolution of 108 × 108 μm, field size of 140 mm × 210 mm, and scan time of 5 min. Bone mineral density (BMD) was analyzed using the InAlyzer software program.

### 2.13. Histological Analysis

For histological analysis, the rat femur was fixed in 10% neutral buffered formalin, decalcified in 10% ethylene diamine tetraacetate, and embedded in paraffin. Paraffin-embedded tissues were sliced into sections and stained with hematoxylin and eosin (H&E). The sections were observed under a DM750 reverse phase microscope (Leica Microsystems, Wetzlar, Germany).

### 2.14. Extraction of Surface Proteins from P. freudenreichii MJ2

Surface proteins *of P. freudenreichii* MJ2 were extracted using a chaotropic reagent as described previously [29,30]. *P. freudenreichii* MJ2 was cultured for 48 h at 30 °C, harvested by centrifugation at 3000 rpm for 10 min, and then washed twice with PBS. Then, 5 M guanidine hydrochloride was added to the bacterial cell pellet at an OD_600_ value of 20 and gently suspended. The suspension was incubated at 50 °C for 15 min. Then, centrifugation at 21,000× *g* for 20 min was performed and the supernatants were collected and dialyzed against PBS for 24 h at 4 °C using a 10 kDa cutoff Slide-A-Lyzer^®^ Dialysis Cassette (Thermo Scientific). The remnant pellets were also collected, diluted to 1 × 10^9^ cells/mL, and dialyzed. After 24 h, the solution in the cassette was collected and the protein concentration was measured by the Bradford assay and then stored at −80 °C.

### 2.15. Statistical Analysis

In vitro experimental values are expressed as the mean ± standard deviation (SD) and in vivo experimental values are expressed as the mean ± standard error of the mean (SEM). Statistical analyses were performed using SPSS 24.0 (SPSS Inc., Chicago, IL, USA). The difference between two groups was tested by the Student’s *t*-test and differences among groups were determined by one-way analysis of variance (ANOVA) followed by Tukey’s honestly significant difference (HSD) post hoc test. A *p*-value of <0.05 was considered statistically significant.

## 3. Results

### 3.1. Cytotoxicity of hkMJ2 in hFOB 1.19 Cells

**Figure 1:**
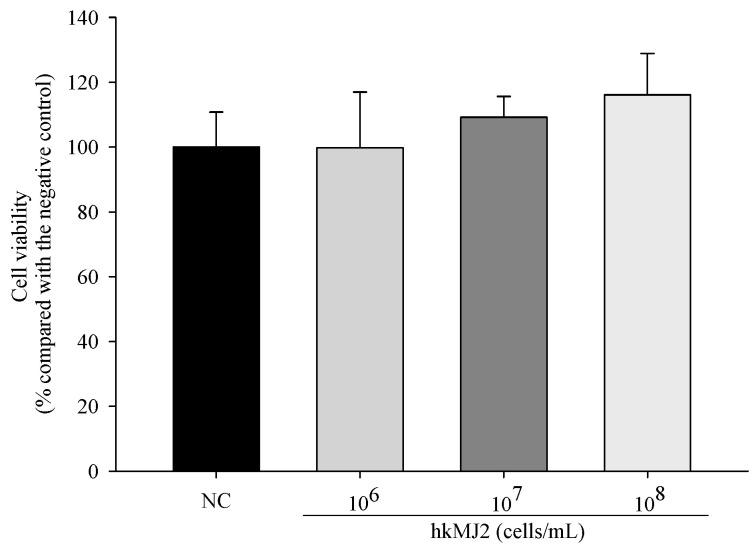
Cytotoxic effect of heat-killed *P. freudenreichii* MJ2 (hkMJ2) on hFOB 1.19 cells. The values indicate the mean ± SD of three independent experiments performed in triplicate.

To investigate the cytotoxicity of hkMJ2 in hFOB 1.19 cells, their viability was measured by the MTT assay. The viabilities of cells treated with hkMJ2 (1 × 10^6^, 1 × 10^7^, or 1 × 10^8^ cells/mL) did not show a significant reduction (Figure 1). Therefore, we used hkMJ2 at concentrations of 1 × 10^6^, 1 × 10^7^, and 1 × 10^8^ cells/mL in this study.

### 3.2. Increase in ALP Activity and Osteoblast Mineralization Induced by hkMJ2

**Figure 2:**
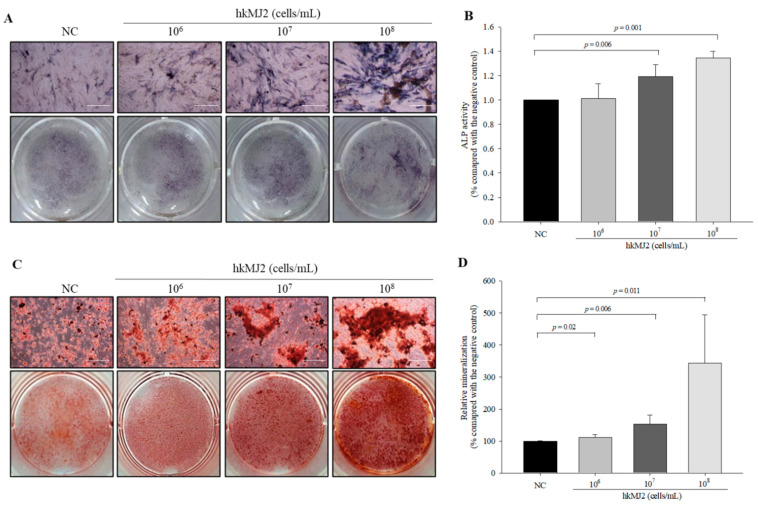
Effect of heat-killed *P. freudenreichii* MJ2 (hkMJ2) on ALP activity and osteoblast mineralization. ALP activity was analyzed by staining with p-nitrophenyl-phosphate (×100, scale bar = 100 μm) (**A**) and quantified (**B**). Calcium deposits were stained by alizarin red S (×100, scale bar = 100 μm) (**C**) and quantified (**D**). Values indicate the mean ± SD of three independent experiments performed in triplicate.

To investigate the effect of hkMJ2 on osteoblast differentiation, we analyzed ALP activity, a relatively early stage marker of osteoblast differentiation. Although ALP activity in cells treated with 1 × 10^6^ cells/mL hkMJ2 did not increase significantly, ALP activities in cells treated with 1 × 10^7^ and 1 × 10^8^ cells/mL hkMJ2 were increased significantly compared with the negative control (Figure 2A,B). These results suggested that hkMJ2 stimulated induction of osteoblast differentiation. To investigate whether hkMJ2 induced terminal osteoblast differentiation, the degree of mineralization was measured using alizarin red S that forms a red complex with calcium. Formation of red deposits was increased significantly in a dose-dependent manner with hkMJ2 compared with the negative control (Figure 2C,D). These results suggested that hkMJ2 increased osteoblast mineralization.

### 3.3. Expression of Osteoblast Differentiation-Related Genes and Proteins Induced by hkMJ2 Treatment

**Figure 3:**
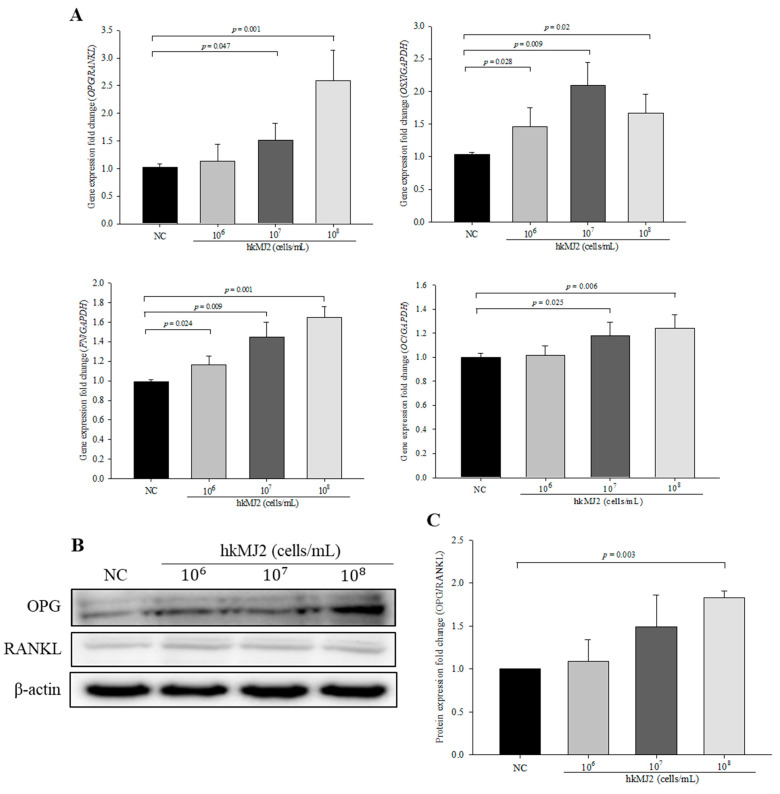
Expression levels of osteoblast differentiation-related genes and proteins in cells treated with heat-killed *P. freudenreichii* MJ2 (hkMJ2). The expression levels of genes were measured by qPCR (**A**) and proteins were measured by Western blotting (**B**) and quantified (**C**). The values indicate the mean ± SD of three independent experiments performed in triplicate.

To investigate the effect of hkMJ2 on the expression levels of genes and proteins related to osteoblast differentiation, we performed qPCR and Western blots, respectively. Although the expression level of osterix (*OSX)* did not change in a dose-dependent manner, it was significantly increased in cells treated with hkMJ2 compared with the negative control. The expression levels of osteogenic genes including *OPG/RANKL*, fibronectin (*FN*), and osteocalcin (*OC*) were significantly increased in cells treated with hkMJ2 in a dose-dependent manner compared with the negative control (Figure 3A). However, although the OPG/RANKL protein ratio in cells treated with hkMJ2 increased gradually as the dose was increased, the OPG/RANKL ratio was significantly increased only in cells treated with 1 × 10^8^ cells/mL hkMJ2 (Figure 3B,C).

### 3.4. 2-DE and Protein Identification

**Figure 4:**
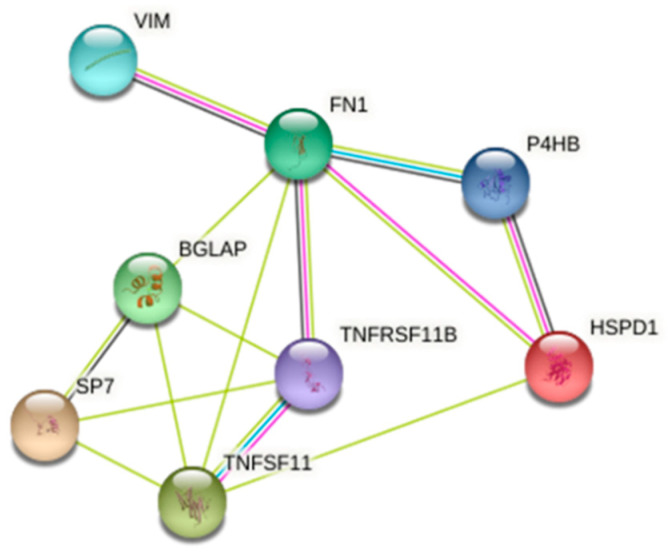
STRING analysis of protein interaction networks. Interactions of identified proteins were mapped by searching STRING database version 10.5 with a confidence cutoff of 0.4. In the resulting protein association network, proteins are presented as nodes connected by lines whose thickness represents the confidence level. Osteoblast differentiation-related genes BGLAP (OC), TNFRSF11B (OPG), SP7 (OSX), TNFSF11 (RANKL), and FN1 (fibronectin) were obtained from the qPCR data (Figure 3).

To explore the possible mechanism of *P. freudenreichii* MJ2 in osteoblast differentiation, we performed 2-DE. Among 690 spots, 51 spots showed increases in the expression level to more than 2-fold and 90 spots showed decreases in the expression level to below 0.5-fold compared with the negative control. Among them, two spots (<0.2-fold) and four spots (>4-fold) were identified by LC-MS/MS (Table 3). To investigate the relationship between these proteins and osteoblast differentiation-related genes that were analyzed in Figure 3, we performed STRING analysis. When the minimum required interaction score was set to a medium confidence of 0.400, 60 kDa heat shock protein (HSPD1), protein disulfide isomerase (P4HB), and vimentin (VIM) showed interactions with osteoblast differentiation-related genes (Figure 4). These results showed that VIM, HSPD1, and P4HB might be related to the osteoblast differentiation-related proteins. In particular, protein disulfide isomerase is related to endoplasmic reticulum (ER) stress and the signaling induced by ER stress has a relationship with bone formation [31,32]. The bone morphogenetic protein 2 (BMP2) signaling pathway mediated by ER stress induces osteoblast differentiation. Therefore, hkMJ2 might play a role in BMP2 signaling related to ER stress and osteoblast differentiation.

### 3.5. hkMJ2 Treatment Activates the BMP2/RUNX2 Signaling Pathway

**Figure 5:**
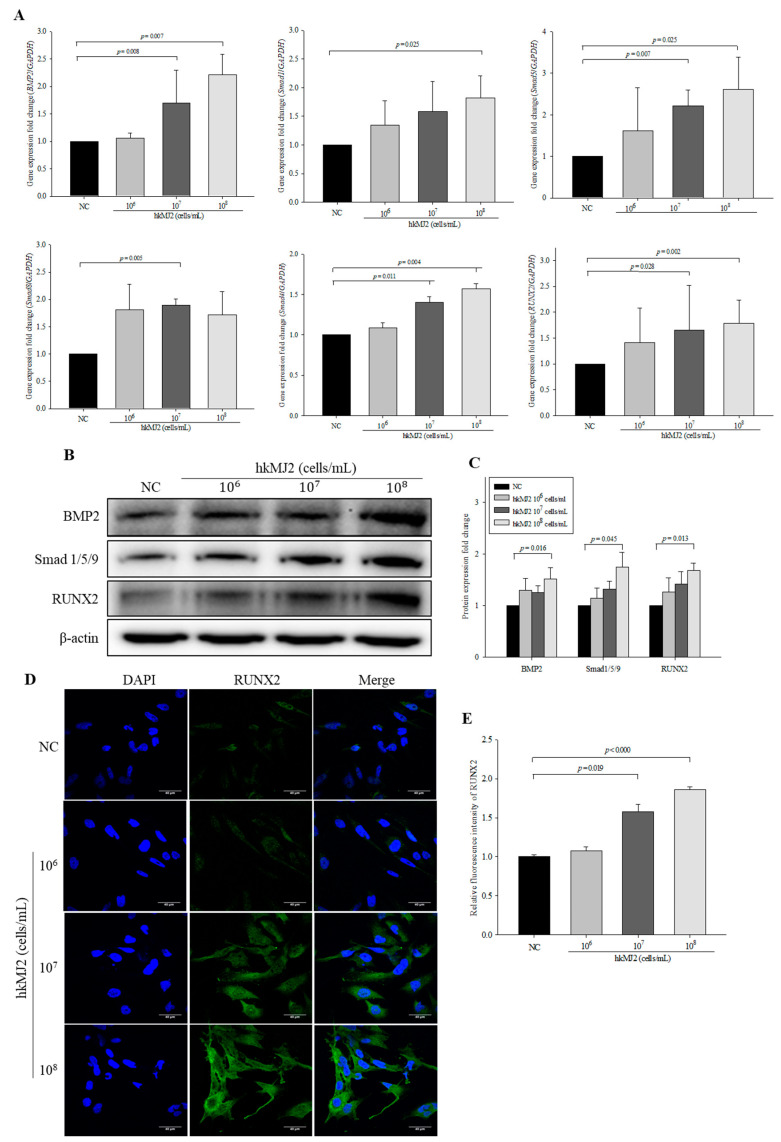
Effect of heat-killed *P. freudenreichii* MJ2 (hkMJ2) on the expression levels of BMP2/RUNX2 signaling-related genes and proteins. The expression levels of genes were measured by qPCR (**A**) and proteins were measured by Western blotting (**B**) and quantified (**C**). The expression level of RUNX2 protein was measured by immunocytochemistry (ICC) (×60, scale bar = 40 μm) (**D**) and the relative intensity was quantified (**E**). The values indicate the mean ± SD of three independent experiments performed in triplicate.

The BMP2 signaling pathway is a crucial mechanism of osteoblast differentiation, followed by activation of RUNX2, a critical transcription factor of osteoblastic differentiation. To investigate the mechanism of hkMJ2-induced osteoblast differentiation, the mRNA expression levels of *BMP2*, *RUNX2*, *Smad1*, *Smad5*, *Smad8*, *Smad4*, and *RUNX2* were analyzed by qPCR. Expression levels of BMP2/RUNX2 signaling pathway-related genes were significantly increased in cells treated with 1 × 10^7^ and 1 × 10^8^ cells/mL hkMJ2 compared with the negative control, except for expression of *Smad1* and *Smad8*, which was significantly increased only in cells treated with 1 × 10^8^ and 1 × 10^7^ cells/mL hkMJ2, respectively (Figure 5A). The protein expression levels of BMP2, Smad 1/5/9, and RUNX2 were significantly increased in cells treated with 1 × 10^8^ cells/mL hkMJ2 compared with the negative control (Figure 5B,C). The expression level of RUNX2 measured by ICC showed a significant increase in cells treated with 1 × 10^7^ and 10^8^ cells/mL hkMJ2 (Figure 5D,E). These results showed that hkMJ2 might promote activation of the BMP2/RUNX2 signaling pathway.

**Figure 6:**
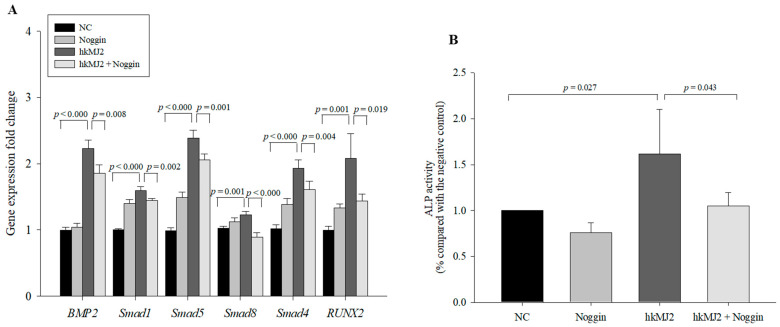
Expression levels of BMP2/RUNX2 signaling-related genes in cells treated with heat-killed *P. freudenreichii* MJ2 (hkMJ2) (1 × 10^8^ cells/mL) and BMP inhibitor Noggin (100 ng/mL) (**A**) and ALP activity (**B**). The values indicate the mean ± SD of three independent experiments performed in triplicate.

To examine whether hkMJ2 enhanced osteoblast differentiation via the BMP2/RUNX2 signaling pathway, we used the BMP inhibitor Noggin [33]. Although cells treated with the inhibitor alone did not show a significant inhibitive effect compared with the negative control, cells cotreated with 100 ng/mL Noggin (and 1 × 10^8^ cells/mL hkMJ2 showed a significant decrease in mRNA expression compared with cells treated with hkMJ2 alone (Figure 6A). Additionally, ALP activity in cells cotreated with Noggin and hkMJ2 showed significant decreases compared with cells treated with hkMJ2 alone (Figure 6B). These results suggest that hkMJ2 promoted osteoblast differentiation via the BMP2/RUNX2 signaling pathway.

### 3.6. Effect of P. freudenreichii MJ2 (MJ2) and/or L. plantarum (LP) Administration to Ovariectomized (OVX) Rats on Body Weight and Hepatotoxicity

To investigate the inhibitory effect of MJ2 on bone loss in the OVX rat model, hkMJ or live MJ2 was administered to the OVX rats and live *L. plantarum* (LP) was used as a reference control. After 16 weeks of treatment, body weights did not show a significant difference among all OVX groups. The OVX groups showed increases in body weights compared with the sham group, which might have been due to the ovariectomy. Additionally, there was no significant difference in glutamic oxaloacetic transaminase (GOT) or glutamic pyruvate transaminase (GPT) levels in serum between the groups (Table 4). These results indicated that live/dead MJ2 and LP administration did not show hepatotoxicity in sham and OVX rats.

### 3.7. Improvement of Femoral BMD and Inhibitory Effect on Bone Loss in MJ2-Treated OVX Rats

**Figure 7:**
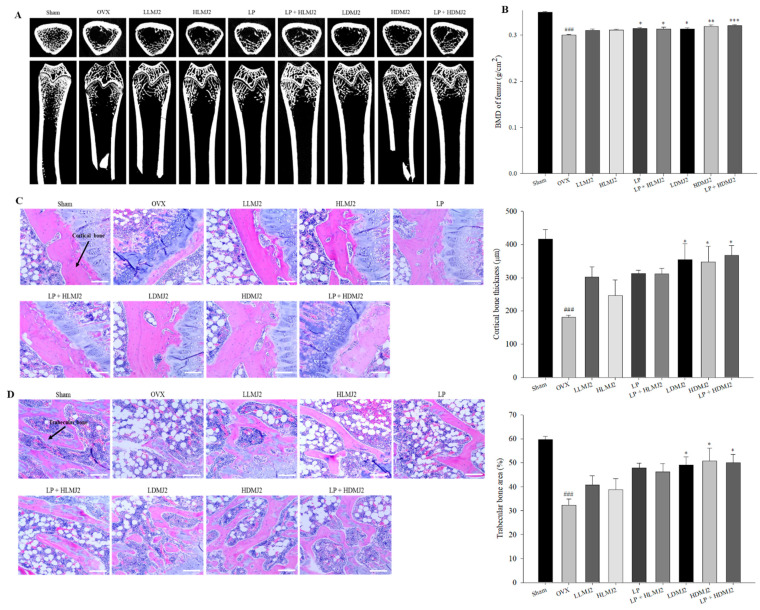
Effect of MJ2 and *L. plantarum* on osteoporotic bone mass and bone microarchitecture. 3D images of the femur of the hind limb were obtained by micro-CT (**A**). Bone mineral density (BMD) measured by dual-energy X-ray absorptiometry scanning was quantified (**B**). Bone microarchitecture (cortical bone (**C**) and trabeculae bone (**D**)) was analyzed by H&E staining and quantified (×100, scale bar = 100 μm). The values indicate the mean ± SEM. ^###^ *p* < 0.001 compared with the sham group; * *p* < 0.05, ** *p* < 0.01, and *** *p* < 0.001 compared with the OVX group.

Micro-CT scan images of the groups are shown in Figure 7A. The BMD of the femur was evaluated by X-ray. The BMD of the OVX group was significantly reduced compared with that of the sham group (Figure 7B). The BMD of bacterium-treated groups, except for groups treated with live MJ, was increased significantly compared with that of the OVX group. These results suggested that dead MJ2 alone or with *L. plantarum* was more effective in preventing bone loss than live MJ2 administration in mice with OVX-induced osteoporosis.

### 3.8. Inhibitory Effect on Bone Loss in MJ2-Treated OVX Rats

To investigate histological changes of femoral tissue, we performed H&E staining. Compared with the sham group, cortical bone in the OVX group had thinned (Figure 7C) and the bone trabeculae area in the OVX group was decreased and ruptured (Figure 7D). Dead MJ2 administration resulted in a marked increase in the cortical bone thickness and area of bone trabeculae. These results suggested that dead MJ2 had an inhibitory effect on bone loss in OVX rats.

### 3.9. MJ2-Treated OVX Rats Show an Increase in the OPG/RANKL Ratio

**Figure 8:**
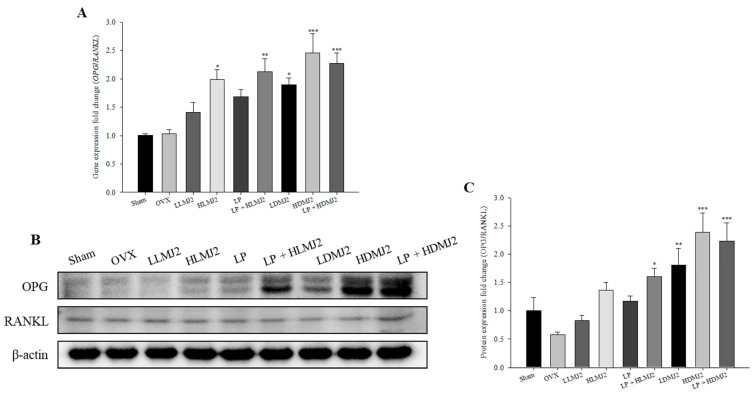
Effect of MJ2 and *L. plantarum* on the OPG/RANKL ratio and expression of osteoblast differentiation-related genes and proteins. The gene expression levels of *OPG/RANKL* (**A**) and expression levels of the proteins were measured by Western blotting (**B**) and quantified (**C**). Each value indicates the mean ± SEM of three independent experiments performed in triplicate. * *p* < 0.05, ** *p* < 0.01, and *** *p* < 0.001 compared with the OVX group.

Gene and protein expression levels of OPG/RANKL did not significantly decrease in the OVX group compared with the sham group, whereas dead MJ2-treated groups showed significant increases in a dose-dependent manner compared with the OVX group (Figure 8).

### 3.10. Viability of Cells Treated with Surface Proteins (SP) Extracted from MJ2

**Figure 9:**
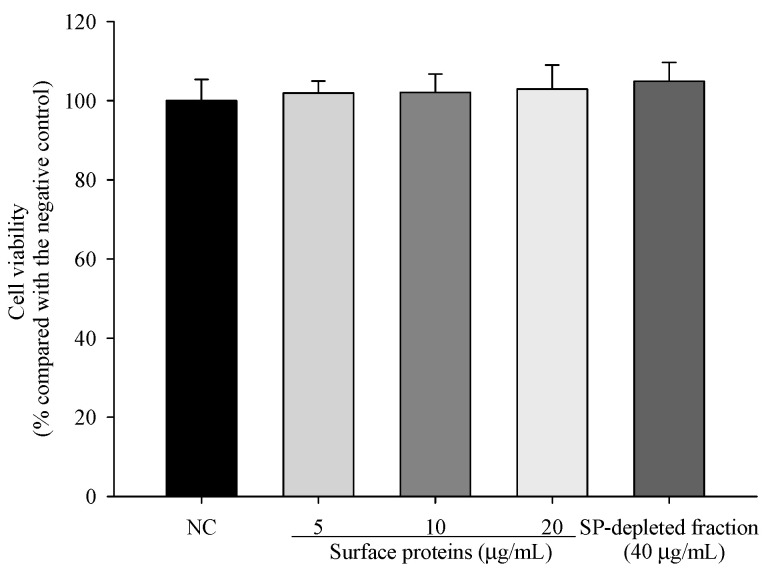
Cytotoxicity of surface proteins (SPs) extracted from MJ2 on hFOB 1.19 cells. The values indicate the mean ± SD of three independent experiments performed in triplicate.

To investigate the cytotoxicity of surface proteins (SPs) and an SP-depleted fraction in hFOB 1.19 cells, cell viability was measured by the MTT assay. The viabilities of cells treated with 5, 10, and 20 μg/mL SPs or the SP-depleted fraction (40 μg/mL) did not show a significant reduction (Figure 9). Therefore, we used these concentrations of SP and the SP-depleted fraction in the following experiments.

### 3.11. ALP Activity and the Expression Levels of Osteoblast Differentiation-Related Genes in Cells Treated with SPs Extracted from MJ2

**Figure 10:**
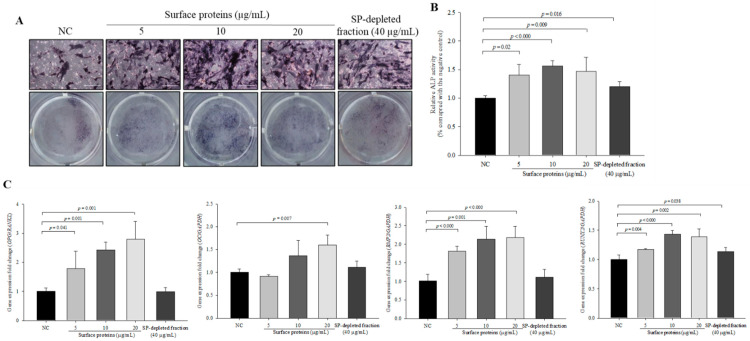
ALP activity and expression levels of osteoblast differentiation-related genes in cells treated with surface proteins extracted from *P. freudenreichii* MJ2. ALP activity was analyzed by staining (×100, scale bar = 100 μm) (**A**) and an activity assay (**B**). The expression levels of osteoblast differentiation-related genes were measured by qPCR (**C**). Values indicate the mean ± SD of three independent experiments performed in triplicate.

To investigate the effect of SPs on early osteoblastic differentiation, we analyzed ALP activity. Relative ALP activities in SP-treated cells were significantly increased compared with the negative control (Figure 10A,B). Unexpectedly, although ALP activity in SP-depleted fraction (40 μg/mL)-treated cells was lower than that in SP-treated cells, ALP activity in the SP-depleted fraction was also significantly increased compared with the negative control. Additionally, the SP-depleted fraction did not increase the mRNA expression levels of osteoblast differentiation-related genes except for *RUNX2*. Expression levels of *OPG/RANKL*, *BMP2*, and *RUNX2* in cells treated with SPs were significantly increased in a dose-dependent manner compared with the negative control except for *OC* that was significantly increased only in 20 µg/mL SP-treated cells (Figure 10C). The SP-depleted fraction did not increase the mRNA expression levels of osteoblast differentiation-related genes except for *RUNX2*. The results suggested that *P. freudenreichii* MJ2-derived SPs were involved in osteoblast differentiation.

### 3.12. Osteoblast Mineralization in Cells Treated with SPs Extracted from MJ2

**Figure 11:**
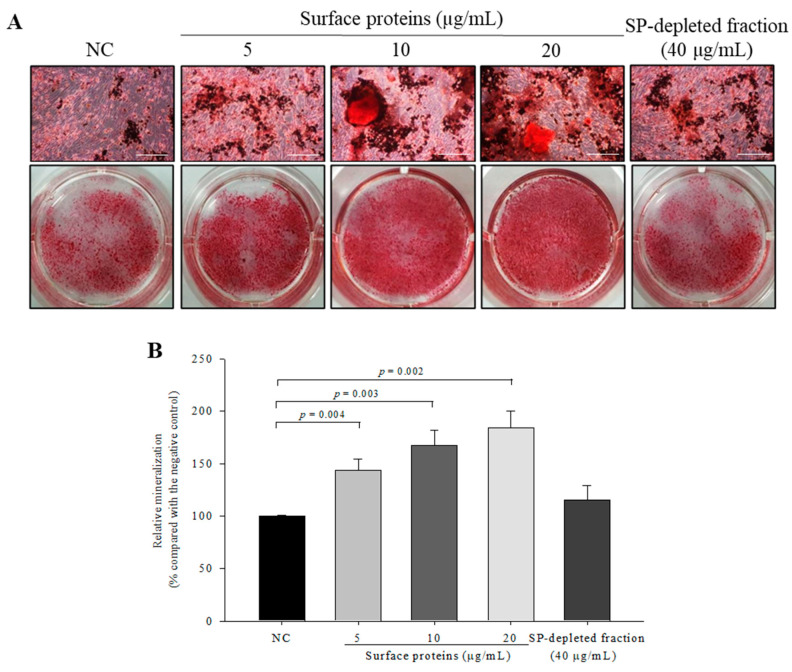
Effect of surface proteins extracted from *P. freudenreichii* MJ2 on osteoblast mineralization. Calcium deposits were stained by alizarin red S (×100, scale bar = 100 μm) (**A**) and quantified (**B**). Values indicate the mean ± SD of three independent experiments performed in triplicate.

To investigate the effect of SPs on osteoblast mineralization, alizarin red S-calcium complex formation was measured by alizarin red S staining. Mineralization in SP-treated osteoblasts was significantly increased in a dose-dependent manner compared with the negative control (Figure 11). However, the SP-depleted fraction did not enhance the osteoblast mineralization. These results showed that *P. freudenreichii* MJ2-derived SPs stimulated the late stage of osteoblast differentiation and increased osteoblast mineralization.

## 4. Discussion

Osteoporosis is a typical disease that commonly occurs in the elderly. Osteoporosis-induced fractures cause physical pain and are costly because of long-term treatment. The collapse of the balance between bone formation by osteoblasts and bone destruction by osteoclasts leads to various bone disorders including not only osteoporosis, but also rheumatoid arthritis and osteopenia [6,34]. Thus, it is important to develop new agents that effectively enhance bone health and are reliable in the long term. Recently, many studies have demonstrated that several probiotics and their metabolites can treat osteoporosis by enhancing bone formation or affecting bone metabolism [16,35,36,37]. *Bifidobacterium longum* increases BMD and bone formation parameters, and *Lactobacillus* strains decrease bone loss in ovariectomized animal models. Butyrate produced by *Lactobacillus rhamnosus* stimulates bone formation by regulating bone anabolism. On the basis of the relationship between gut microbiota and osteoimmunology, the effects of probiotics on bone health have been studied [38]. The definition of probiotics by the WHO is living microorganisms that have health benefits. Thus, most studies of probiotics have been performed with live probiotics. Inactivated probiotics have shown similar beneficial effects to live probiotics in vitro [39]. However, there has been little information on the effect of dead probiotics on bone formation and its mechanism. In this study, hkMJ2 showed an enhancing effect on bone formation.

ALP activity is a phenotypic marker for early stage osteoblast differentiation and increases the expression levels of osteoblast differentiation-related genes and proteins. hkMJ2 significantly increased ALP activity, which indicates that hkMJ2 stimulates the early stage of osteoblast differentiation. The OPG/RANKL ratio is a crucial indicator of osteoblast and osteoclast-related differentiation. OPG enhances osteoblast differentiation and inhibits osteoclast differentiation by blocking RANK and RANKL binding [40]. When RANK on osteoclast precursors and RANKL bind as the initial signal, osteoclast formation and differentiation follow. However, OPG inhibits their interaction by binding to RANKL instead of RANK. hkMJ2 increased the OPG/RANKL ratio, which indicates that hkMJ2 increases osteoblast differentiation and inhibits osteoclast differentiation. OSX is expressed in all types of developing bone cells and inhibits differentiation of progenitor cells to chondrocytes [41]. FN is a large glycoprotein that binds to cells and engages in cellular processes including wound healing, cell migration, and cytoskeleton organization [42], which also contributes to osteoblast survival and differentiation. OC, as a type of bone non-collagenous protein, is synthesized at the late stage of osteoblast differentiation [43]. hkMJ2 increased the expression levels of OSX, FN, and OC, which suggests that hkMJ2 stimulates osteoblast differentiation by upregulating genes related to the osteoblast differentiation process. OC is a hallmark of terminal osteoblast differentiation. Mineralization is considered as an indicator of successful osteoblast differentiation. Alizarin red s–calcium complex formation increased in cells treated with hkMJ2, which suggests that hkMJ2 enhanced calcium deposits in the differentiated osteoblasts. Therefore, hkMJ2 stimulated osteoblast differentiation by stimulating gene expression at the early differentiation stage and accomplished osteoblast differentiation, which increased osteoblast mineralization.

Among the proteins identified by 2-DE and LC-MS/MS in cells treated with hkMJ2, HSPD1 and P4HB are associated with osteoblast differentiation-related proteins. HSPD1 is a mitochondrial chaperonin protein that regulates homeostasis of mitochondrial functions and maintains protein-folding processes. P4HB is a multifunctional enzymatic protein involved in the cellular ER stress response and also acts as a molecular chaperone. STRING generates a network of protein interactions from high-throughput experimental data and predictions on the basis of genomic context analysis. STRING analysis of the gene expression results and identified proteins showed that hkMJ2 stimulated osteoblast differentiation. β-actin (ACTB) and vimentin (VIM) are critical components of the cytoskeleton and these two proteins were increased in cells treated with hkMJ2. VIM is predominantly expressed in cells of mesenchymal origin, which include osteoblasts. VIM has been reported to inhibit activating transcription factor 4 (ATF4)-mediated OC transcription and delay osteoblast differentiation [44]. Osteoblast differentiation is mediated by several osteoblast-specific transcription factors such as OSX and ATF4. Interestingly, in this study, both VIM expression and *OC* gene expression were increased in cells treated with hkMJ2. In particular, hkMJ2 increased RUNX2 expression at transcriptional and translational levels, which suggests that hkMJ2 increases osteoblast differentiation via the RUNX2 transcription factor and not ATF4. BMP2, which belongs to the TGF-β superfamily, stimulates bone formation by inducing osteoblast differentiation [31,32]. It interacts with a receptor and then induces signal transduction through activation of SMAD proteins that activate SMAD1, SMAD5, or SMAD8. Ultimately, this process promotes transcription of osteoblastic genes, which enhances the mineralization of mature osteoblasts [41]. A stress-induced heat shock protein enhances bone formation by activating BMP signaling [45] and BMP2 signaling increases RUNX2-induced osteoblast differentiation through mild ER stress. In this study, hkMJ2 increased the gene and protein expression levels of BMP2, Smad1/5/8, and RUNX2. Additionally, BMP inhibitor Noggin suppressed the promoting effect of hkMJ2 on osteoblast differentiation. These results suggest that hkMJ2 promoted osteoblast differentiation through the BMP2/RUNX2 signaling pathway.

Ovariectomy is a typical procedure to induce postmenopausal osteoporosis in an animal model. In this study, ovariectomy-induced estrogen deficiency decreased BMD, and heat-killed dead MJ2 and *L. plantarum* (LP) significantly increased BMD of the femur, whereas, unexpectedly, live MJ2 did not increase BMD. In particular, unlike live MJ2 and LP, dead MJ2 administration led to a significant increase in cortical bone thickness and the trabecular bone area. These results suggest that dead MJ2 is more effective for the prevention of bone loss in OVX rats than live MJ2. In addition to the increase in BMD, dead MJ2 might be more advantageous for manufacturing to improve bone diseases such as osteoporosis than live MJ2 because of the longer shelf life of dead MJ2. Additionally, dead MJ2 is more effective for the prevention of bone loss in OVX rats than LP that has anti-osteoporotic effects [28]. LP alleviates osteoporosis by suppressing NF-κB-linked TNF-α expression and alleviates gut inflammation, which improves osteoporosis. Unexpectedly, cotreatment with live or dead MJ2 and LP did not show a synergetic effect in OVX rats. The OPG/RANKL ratio at transcriptional and translational levels showed consistency with the BMD in OVX rats treated with dead MJ2 except for the high dose of live MJ2, which significantly increased gene expression of the OPG/RANKL ratio. Therefore, dead MJ2 might inhibit osteoporotic bone loss by promoting osteoblast differentiation. In our next study, induction of gut microbiota homeostasis by MJ administration should be investigated in OVX rats with a disrupted composition of gut microbiota.

Bacterial surface proteins are displayed in the surface-exposed cell wall. In Gram-positive bacteria, surface proteins are classified in accordance with covalent and non-covalent interactions [46]. Covalently attached proteins include proteins anchored to the cytoplasmic membrane, lipoproteins anchored to the membrane lipid layer, and proteins attached to peptidoglycans by sortase. Non-covalently attached proteins include binding via cell wall-binding domains such as the choline-binding domain, lysin motif domain, and S-layer homology motif. Additionally, there are other extractable surface-bound proteins referred to as moonlighting proteins that are originally in the cytoplasm, but located on the cell surface through an unknown mechanism [47]. In the case of probiotics, their surface proteins are known to play various roles in stress tolerance, adhesion to mucus, and immunomodulation. Surface proteins extracted from *P. freudenreichii* ITG P20 have an anti-inflammatory activity by regulating cytokine induction [30]. In this study, extracted surface proteins increased ALP activity, mRNA expression levels of osteoblast differentiation-regulated genes, and osteoblast mineralization. In vitro and in vivo experiments showed that hkMJ2 significantly increased osteoblast differentiation and mineralization as well as BMD. Therefore, the effective protein(s) among the surface proteins might be resistant to heat or denatured protein(s) might have an increased effect on osteoblast differentiation and mineralization.

## 5. Conclusions

*P. freudenreichii* MJ2 isolated from raw milk increases osteoblast differentiation and mineralization in hFOB 1.19 human fetal osteoblastic cells. In particular, hkMJ2 is more effective than live MJ2. Specifically, surface proteins of MJ2 promote osteoblast differentiation and mineralization via the BMP2/RUNX2 signaling pathway. hkMJ2 also increases BMD of OVX rats. Therefore, these results suggest that hkMJ2 can be used to improve bone health and treat osteoporosis.

## Figures and Tables

**Table 1:** Primer sequences used for qPCR.

Gene	Species	Forward (5′–3′)	Reverse (5′–3′)
*GAPDH*	Human	GAG TCA ACG GAT TTG GTC GT	GAC AAG CTT CCC GTT CTC AG
*BMP-2*	Human	CCC AGC GTG AAA AGA GAG AC	GAG ACCGCAGTC CGT CTA AG
*Smad1*	Human	GGT TCC AAG ACA CAG CGA AT	TGG GAG AGT GAG GGT AGG TG
*Smad5*	Human	AAC CTG AGC CAC AAT GAA CC	GTG GCA TAT AGG CAG GAG GA
*Smad8*	Human	CCA CAG AAG CCT CTG AGA CC	CCC AAC TCG GTT GTT CAG TT
*Smad4*	Human	AAA GGT GAA GGT GAT GTT TGG GTC	CTG GAG CTA TTC CAC CTA CTG ATC C
*RUNX2*	Human	GCA GAC AGC TCA CAA AAC CA	GGA AAA GGG GAG AAG GAG TG
*OSX*	Human	GCT TAT CCA GCC CCC TTT AC	CAC TGG GCA GAC AGT CAG AA
*FN*	Human	AAT CCA AGC GGA GAG AGT CA	CAT CCT CAG GGC TCG AGT AG
*OPG*	Human	TGA GGA GGC ATT CTT CAG GT	CGC TGT TTT CAC AGA GGT CA
*RANKL*	Human	AGG CCT TTC AAG GAG CTG TG	TTG GAG ATC TTG GCC CAA CC
*OC*	Human	CCC GAA GGA GCT GAG GAC AC	CTT TGA CCC TGC TTC CAG AG
*Β-actin*	Rat	AGC CAT GTA CGT AGC CAT CC	CTC TCA GCT GTG GTG GTG AA
*OPG*	Rat	TGG GAA TGA AGA TCC TCC AG	GAG GAA GGA AAG GGC CTA TG
*RANKL*	Rat	CAT GAA ACC TCA GGG AGC GT	GTT GGA CAC CTG GAC GCT AA

**Table 2:** Animal groups used in this study.

Group	Ovariectomy (OVX)	Treatment	Abbreviation
1	Sham	Phosphate buffered saline (PBS)	Sham
2	OVX	PBS	OVX
3	OVX	Low-dose live *P. freudenreichii* MJ2 (1 × 10^7^ CFU/mL)	LLMJ2
4	OVX	High-dose live *P. freudenreichii* MJ2 (1 × 10^8^ CFU/mL)	HLMJ2
5	OVX	Live *L. plantarum* (1 × 10^8^ CFU/mL)	LP
6	OVX	Live *L. plantarum* + high-dose live *P.* *freudenreichii* MJ2	LP + HLMJ2
7	OVX	Low-dose dead *P. freudenreichii* MJ2 (1 × 10^7^ cells/mL)	LDMJ2
8	OVX	High-dose dead *P. freudenreichii* MJ2 (1 × 10^8^ cells/mL)	HDMJ2
9	OVX	Live *L. plantarum* + high-dose dead *P.* *freudenreichii* MJ2	LP + HDMJ2

**Table 3:** Proteins identified by two-dimensional electrophoresis (2-DE) and LC-MS/MS.

Protein Name	Expression Change (Fold) ^1^	*gi* Number
Putative, partial	0.125	*gi*:553734
Survival/evasion peptide (DCD)	0.161	*gi*:15375076
60 kDa heat shock protein (HSPD1)	4.326	*gi*:129379
Chain A, protein disulfide isomerase (P4HB)	7.752	*gi*:159162689
Vimentin (VIM)	16.971	*gi*:340219
Mutant beta-actin (ACTB)	4.096	*gi*:283336

^1^ Fold change of differently expressed proteins in the 1 × 10^8^ cells/mL hkMJ2-treated group versus the control group.

**Table 4:** Body weights and serum levels of glutamic oxaloacetic transaminase (GOT) and glutamic pyruvate transaminase (GPT) of ovariectomized (OVX) rats.

Group	Body Weight (g)	GOT (U/L)	GPT (U/L)
Sham	333.58 ± 5.73	87.0 ± 7.39	32.8 ± 3.86
OVX	401.03 ± 8.20	110.1 ± 8.69	52.5 ± 7.58
LLMJ2	401.71 ± 5.25	89.6 ± 11.18	41.6 ± 6.81
HLMJ2	388.79 ± 9.33	92.0 ± 14.13	33.8 ± 3.81
LP	391.67 ± 6.96	75.6 ± 5.20	30.4 ± 2.22
LP + HLMJ2	373.54 ± 7.34	87.4 ± 15.94	36.5 ± 8.85
LDMJ2	378.84 ± 5.37	73.4 ± 2.66	33.3 ± 1.82
HDMJ2	372.51 ± 4.45	67.1 ± 3.81	30.5 ± 1.36
LP + HDMJ2	396.98 ± 11.31	80.9 ± 14.14	39.8 ± 8.53

Values indicate the mean ± SEM.

## Data Availability

The information on the data utilized for analysis is provided in the Methods section of this manuscript.
